# Proteomic Characterization of the Rhesus Macaque Lens Nucleus: Similarity to Human Lens, Age Effects on Protein Solubility, and Trends in Post-Translational Modifications

**DOI:** 10.1167/iovs.66.12.28

**Published:** 2025-09-12

**Authors:** Billy L. Hayden, Owen Kelley, Keith Zientek, Ashok P. Reddy, Phillip A. Wilmarth, Rachel Munds, Michael J. Montague, Melween I. Martinez, Gadi Wollstein, James P. Higham, Arturo O. Barron Arrambide, John Danias, Amanda D. Melin, Larry L. David, Jeremy A. Whitson

**Affiliations:** 1 Department of Biology, High Point University, High Point, North Carolina, United States; 2 Proteomics Shared Resource, Oregon Health & Science University, Portland, Oregon, United States; 3 Department of Anthropology and Archaeology, University of Calgary, Calgary, Alberta, Canada; 4 Department of Neuroscience, University of Pennsylvania, Philadelphia, Pennsylvania, United States; 5 Cayo Biobank Research Unit, Department of Neuroscience, University of Pennsylvania, Philadelphia, Pennsylvania, United States; 6 Caribbean Primate Research Center, University of Puerto Rico, San Juan, Puerto Rico, United States; 7 Advanced Center of Ophthalmic Research in Neuroimaging, Wills Eye Hospital, Philadelphia, Pennsylvania, United States; 8 Center for the Study of Human Origins, Department of Anthropology, New York University, New York, New York, United States; 9 Department of Ophthalmology, SUNY Downstate Health Sciences University, Brooklyn, NY, United States; 10 Department of Medical Genetics, University of Calgary; Alberta Children's Hospital Research Institute, University of Calgary, Calgary, Alberta, Canada

**Keywords:** nonhuman primate (NHP), aging, lens, proteomics

## Abstract

**Purpose:**

Proteomes of lens nuclei from young (4 years old) and old (15–16 years old) rhesus macaques (*Macaca mulatta*) were analyzed to determine similarity of the proteomic profile to that of human lenses, age-related differences in protein solubility, and association of various post-translational modifications with age and protein solubility.

**Methods:**

Lens core proteins were separated into water-soluble and water-insoluble fractions using aqueous buffer and centrifugation. The water-insoluble fraction was solubilized using sodium dodecyl sulfate (SDS). Proteins were processed using S-trap columns, and peptide digests were analyzed using high-resolution, label-free data-dependent acquisition (DDA) proteomics. Open modification searches were performed using MSFragger to identify possible post-translational modifications (PTMs). The number of modified peptide tandem mass spectra confidently assigned to samples by age or solubility were compared to find PTMs with statistically significant count differences.

**Results:**

The overall proteomic profile of rhesus macaque lenses was very similar to human lenses, consisting of 80.2% crystallins, 1.1% beaded filament proteins, and 18.7% other proteins. The crystallin fraction consisted of 27% alpha crystallins, 67.6% beta/gamma crystallins, and 5.4% taxon-specific psi crystallin. Glycolytic enzymes, beta/gamma crystallins, and a few glutathione-related enzymes were found to have age-related shifts to the water-insoluble fraction. There were significant differences in deamidation, dioxidation, carbamylation, carboxymethylation, and trioxidation based on age and/or solubility of proteins.

**Conclusions:**

These data indicate a high level of conformity between rhesus macaque and human lens proteomes, and a few key differences. We identified several age-related differences in protein solubility and PTM that may contribute to lens pathology.

Age-related nuclear cataract is a major disease of the lens and one of the leading causes of blindness globally; comprising nearly 40% of all cases of moderate and severe vision impairment.^1^ Age-related nuclear cataract pathology is driven by the accumulation of nonenzymatic post-translational modifications (PTMs) on lens proteins, including oxidation, deamidation, and several other PTMs. This alteration to protein structure can lead to loss of protein solubility, protein aggregation, and opacities.^2^^–^^4^ This is especially notable in the lens nucleus, where proteins experience negligible turnover throughout the entire life of an organism.^5^ Further characterization of the variety, frequency, and impact of lens PTMs provides insights into the mechanisms underlying cataractogenesis and may identify novel therapeutic targets for preventative treatments.

Although many studies have analyzed the proteome of human and rodent lenses,^6^^–^^8^ many fewer studies have utilized nonhuman primate (NHP) lenses for this purpose. The NHPs have several potential advantages compared with rodents when used as animal models for lens research. Primates are evolutionary much closer to humans and live longer lives compared with most rodent models. Like humans, they are also diurnal, whereas commonly used rodent models are nocturnal. This is significant because a major source of nonenzymatic PTMs found in the human lens is UV-induced photooxidation.^9^^,^^10^

Rhesus macaque (*Macaca mulatta*) eyes show many similar age-related physiological alterations to those found in human eyes, including increased intraocular pressure, decreased corneal thickness, decreased retinal ganglion thickness, myopic shift, macular legions, and development of nuclear cataract.^11^^–^^13^ Additionally, rhesus macaque lenses are a well-established model for the study of presbyopia.^14^ One known difference between the human and macaque lens is the presence of high levels of betaine-homocysteine S-methyltransferase 2 (BHMT2) in the latter, which has also been termed psi crystallin.^15^ This represents a taxon-specific lens crystallin that exists in the rhesus macaque lineage but not in humans. However, to date, no studies have been published to characterize the rhesus macaque lens proteome, including age-related differences in protein abundance, solubility, and PTM status. These data are necessary to fully confirm the rhesus macaque lens as an accurate model for the study of cataractogenesis.

In the present study, we analyzed the proteomes of lens nuclei from young (4 years old) and aged (15–16 years old) female rhesus macaques from Cayo Santiago; a small island off the coast of Puerto Rico. This population of NHPs was established in 1938 and is free ranging, with individuals being tracked throughout their life.^16^^,^^17^ Although exhibiting more variability than traditional laboratory animal models, the unique nature of this population provides more relevance to modeling human lens pathology as these animals are subject to stress, disease, scarcity, daily natural light exposure, and other conditions as found in the human population but not in a controlled laboratory environment. Conversely, samples from these animals are still expected to display less biological variation than human samples given that these animals do not receive medications, share the same diet, and live in a uniform environment. This combination of factors indicates that these macaques may be a superior animal model for studying lens pathology.

In addition to comparing across age groups, samples were separated into water soluble (WS) and insoluble (WI) fractions to allow for assessment of the impact of age and PTMs on protein solubility. Results were compared across four groups: young water insoluble (YWI), young water soluble (YWS), old water insoluble (OWI), and old water soluble (OWS). Comparisons were only made across a single variable at a time, either solubility or age, so the four comparisons are: OWI versus OWS, YWI versus YWS, OWI versus YWI, and OWS versus YWS. Our goals were to see if age-related decreases in solubility of cytosolic proteins occurred and if higher levels of damaging nonenzymatic PTMs were associated with loss of solubility and increased age, as has been widely reported in studies of human and mouse lenses.^3^^,^^18^^,^^19^

## Methods

### Tissue Collection

Lenses from 4 young (4 years old) female and 4 aged (14–16 years old) female individuals were sampled from a tissue biobank that includes rhesus macaque samples from 137 animals collected in 2019.^20^ Samples were collected from both sexes with ages spanning from 2 months to 19 years. All tissues were collected from the right eyes.

To collect the lens with minimal damage, the aqueous humor was collected using a sterile syringe. Then, a small incision was made approximately 3 mm behind the limbus and surgical scissors were used to open the eye in an anterior segment comprising the ciliary body, lens, iris, and cornea and a posterior segment comprising the retina, choroid posterior sclera, and optic nerve. The anterior segment was further dissected by first removing the remaining vitreous humor. With the lens exposed, gentle cuts were made to the ciliary zonules (suspensory ligament) holding the lens. A small surgical spatula was used to break any remaining zonules. Once detached, the lens was transferred to a 15 mL tube which was then stored in a −80°C freezer. All instruments used were cleaned with 70% ethanol solution and RNAZap (Thermo Fisher Scientific, Waltham, MA, USA) between collections.

### Lens Coring

Frozen lenses were placed on a dry ice cooled glass dish. A precooled 2.7 mm microtrephine was affixed by applying downward pressure on the lens pole and a precooled single edged razor blade was then used to shave off the equatorial region of the lens using downward pressure around the circumference of the trephine so that only a cylinder remained containing the nucleus and equatorial regions at each pole. This lens cylinder was then rotated 90 degrees and approximately 1.5 mm of a facing pole was removed with the single edged razor blade. The cylinder was then detached from the trephine and placed so that the other pole could be similarly removed while immobilizing the lens cylinder with the cooled trephine, leaving only the nuclear region of the frozen lens.

### Protein Isolation

Then, 1 mL of 20 mM phosphate buffer (pH 7.0) containing 1 mM EDTA, 10 µM trichostatin A, 10 mM nicotinamide, and 1% (v/v) phosphatase inhibitor cocktail (P0044; MilliporeSigma, Burlington, MA, USA) was added to each lens core, followed by homogenization using a pellet pestle. EDTA, trichostatin A, nicotinamide, and phosphatase inhibitor were included to prevent changes to oxidation, acetylation, and phosphorylation, and state of proteins, respectively. These homogenates were then centrifuged at 20,000 × g for 15 minutes at 4°C, and the supernatants removed. The pellets were then washed once by addition of another 1 mL of buffer and vigorous vortexing to resuspend, followed by a second centrifugation step as before. The supernatants were then combined to produce the lens water-soluble fraction. Another 1 mL of the same buffer, but with the addition of 5% (w/v) SDS, was then added to the pellets which were resuspended with vigorous vortexing. This suspension constituted the WI fraction. SDS was also added to the final WS fraction to a final concentration of 5% to match the concentration of the WI fraction. Both WS and WI fractions were then assayed for protein content using a Qubit 3.0 fluorometer (Thermo Fisher Scientific). Then, 75 µg aliquots of each sample were lyophilized and then stored frozen at −80°C.

### Peptide Preparation

Peptides were prepared following the manufacturer's instructions using an S-Trap micro kit (K02MICRO10; Protifi LLC, Fairport, NY, USA) to bind proteins, wash off SDS and other impurities, reduce and alkylate cysteine residues, and trypsin digest the proteins. Tryptic peptide samples were then lyophilized, resuspended in 50% (v/v) aqueous methanol solution to remove all traces of TEAB, lyophilized again, and stored frozen at −80°C.

### Liquid Chromatography/Mass Spectrometry Analysis

The samples were injected onto an Acclaim PepMap 100 µm × 2 cm NanoViper C18, 5 µm trap (Thermo Fisher Scientific) at a 5 µL/minute flow rate on a switching valve. After 10 minutes of loading, the trap column was switched on-line to a PepMap RSLC C18, 2 µm, 75 µm × 25 cm EasySpray column (ThermoFisher Scientific). Peptides from lens digests were then separated using a 7.5% to 30% ACN gradient over 90 minutes in the mobile phase containing 0.1% formic acid at a 300 nL/min flow rate. Full parameters can be found in Supplementary Table S1.

Tandem mass spectrometry data were collected using an Orbitrap Q-Exactive instrument (Thermo Scientific, San Jose, CA, USA) configured with an EasySpray NanoSource. Survey scans were performed in the Orbitrap mass analyzer at resolution = 120,000 from 375 to 1400 m/z. Data-dependent MS2 scans following HCD fragmentation (NCE = 30%) were performed on the top 10 ions in the Orbitrap at resolution 30,000 using auto duration dynamic exclusion. Full instrument parameters are listed in Supplementary Table S2.

### Data Analysis

Proteome UP000006718 (Macaca mulatta, taxon ID 9544) canonical FASTA sequences (21,893 proteins, one protein per gene) were downloaded May 2024 from www.UniProt.org. Common contaminants (175 sequences) were added, and sequence-reversed entries were concatenated for a final protein FASTA file of 44,136 sequences.

The 16 binary instrument files were processed with MSFragger^21^ and FragPipe (https://fragpipe.nesvilab.org). An initial open search^22^ was done and summarized with PTM-Shepherd^23^ to find the most common PTMs present. A second search was performed with a restricted set of common PTMs. MSFragger configuration parameters are listed in detail in a spreadsheet tab in Supplementary Appendix S1. Some important parameters were trypsin strict enzyme choice with up to 2 missed cleavages, parent ion and fragment ion tolerances were 20 PPM (MSFragger recommended setting), a limited number of common variable PTMs were specified (as listed in the Results section), and alkylated cysteine was specified as a static modification. The common PTM search results were processed with FragPipe using Philosopher^24^ to filter PSMs to 1% FDR, infer proteins, and apply additional filtering to 1% FDR the protein level. The common PTM search results were summarized with PTM-Shepherd to compare PTM levels between lens groups.

Protein relative abundances, estimated by total spectral counts (SpC), for the four biological groups (with 4 replicates per group) were compared using the Bioconductor R package edgeR^25^ exact testing with Benjamini-Hochberg multiple testing corrections following trimmed mean of M-values normalization^26^ of the spectral count data. Boxplots before and after the application of normalization can be found in Supplementary Figure S1. The protein data for comparisons was filtered to determine quantifiable proteins by requiring an average spectral count value of 1.0 or greater (average over all 16 samples). This removed non-quantifiable proteins with excessive missing values. A value of 0.25 was added to all corrected SpC values to address any remaining missing values before TMM-normalization and statistical testing. The number of quantifiable proteins was 201.

The mass spectrometry proteomics data have been deposited to the ProteomeXchange Consortium (http://proteomecentral.proteomexchange.org) via the PRIDE partner repository^27^ with the dataset identifier PXD062952.

### Statistical Analysis

Ingenuity Pathway Analysis version 127006219 (QIAGEN, Hilden, Germany) software was used to determine z-scores for canonical pathways.

Statistical significance of global PTM level comparisons across groups was performed using 1-way ANOVA with Tukey correction for multiple comparisons.

## Results

### Relative Protein Abundance Comparison

There were 311,817 acquired MS2 scans from the 16 sample liquid chromatography (LC) runs. There were 132,178 scans passing the 1% FDR filtering (an overall ID rate of 42%). The PSMs mapped to 547 protein groups (520 after excluding common contaminant matches) and 462 proteins had 2 or more mapped PSMs. The average number of proteins identified per sample was 160 ± 40. After filtering out low abundance proteins with excessive missing values, 201 proteins were quantifiable in the statistical comparisons. TMM-normalized SpCs and statistical analysis for the 4 comparisons for all 201 proteins can be found in Supplementary Appendix S1.

**Figure 1.**
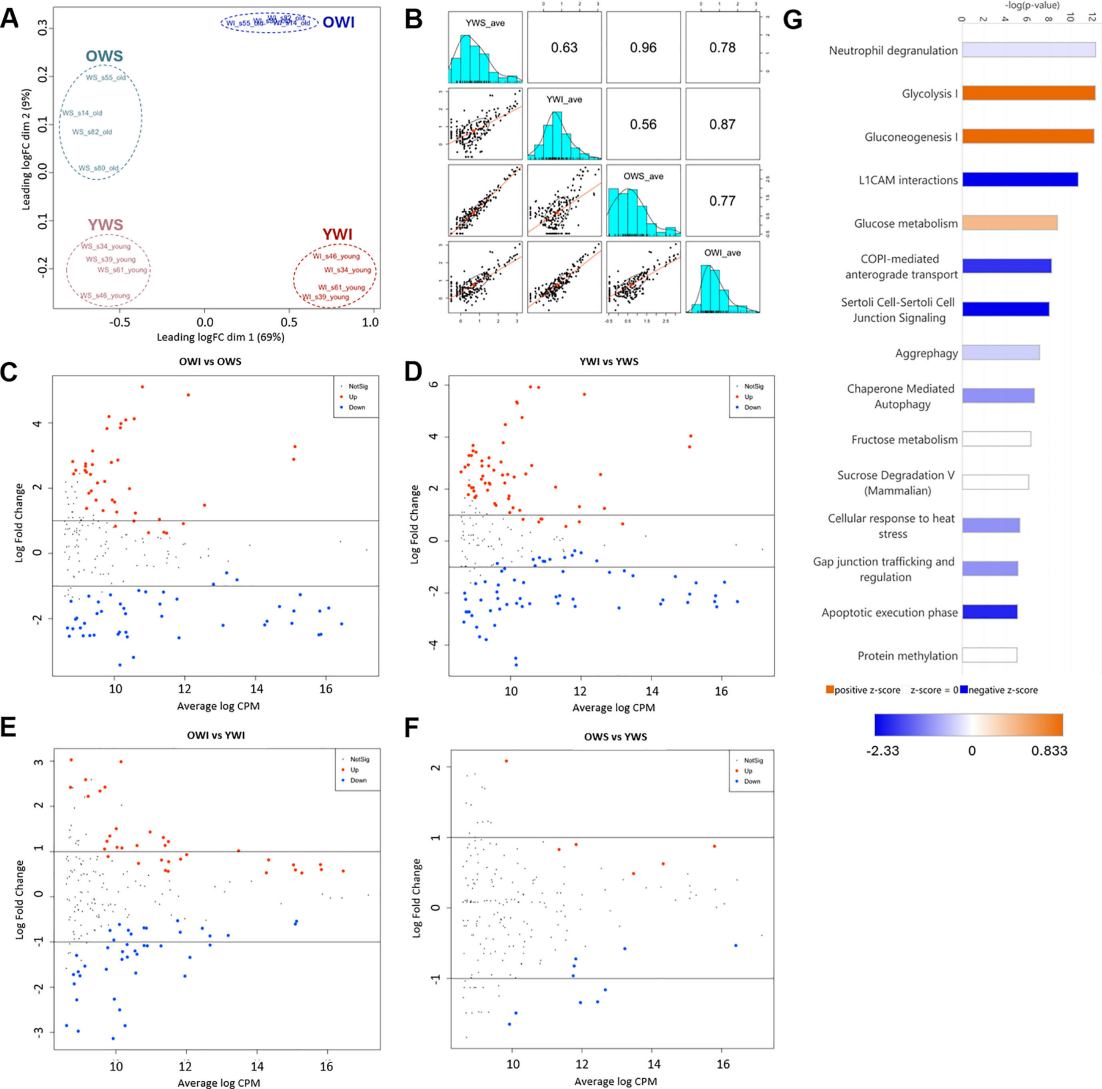
**Overview of protein abundance results.** (**A**) Multi-Dimensional Scaling (MDS) plot from protein abundance data. (**B**) Scatter plot grid of group averages for the 201 quantifiable proteins. (**C–F**) Volcano plots of each comparison. Significantly (adjusted *P* value < 0.05) more abundant proteins are shown in *red*, significantly less abundant proteins are shown in *blue*, and proteins that are not significantly changed are shown as *small black dots*. (**G**) The z-scores of the top 15 impacted canonical pathways in the OWI to YWI comparison based on all protein abundance differences with an adjusted *P* value < 0.05. All groups contained four each.

Multi-dimensional scaling (MDS) indicated that solubility was the primary driver of differential abundance, with a strong separation of WI and WS fractions across dimension 1, whereas age was a secondary factor, with samples segregating by age across dimension 2 (Fig. 1A). Notably, there was a greater separation based on age in the WI fraction compared to the WS fraction, which is also clear when observing the scatter plots (Fig. 1B) and volcano plots (Figs. 1C–F) comparing the group averages for the 201 quantifiable proteins. Analysis of canonical pathway z-scores in the OWI versus YWI comparison (i.e. age-dependent differences in the insoluble fraction) demonstrated a positive z-score (higher in old relative to young) in pathways of glucose metabolism, gluconeogenesis, and glycolysis, whereas all other pathways had a negative or neutral z-score (Fig. 1G).

An analysis of the top 15 (ranked by abundance) relatively more soluble proteins (i.e. the most abundant proteins of the lens fiber cytosol) showed a shift in solubility when comparing across age (Table 1A). CRYBB1, CRYBA1, CRYGC, BHMT2, CRYBA4, CRYGD, SERPINB6, CRYGB, ALDH1A1, CRYGA, PRDX6, and FABP5 all showed a higher percentage of the protein in the WI fraction in old samples compared with young samples, with the OWI versus YWI comparison being statistically significant (adjusted *P* < 0.05) for all of these. Analysis of the top 15 relatively more insoluble proteins (i.e. proteins that are normally found primarily in the WI fraction at any age, such as membrane-bound proteins) showed the opposite trend, with BFSP2, BFSP1, PLEC, GJA8, SLC2A1, NRCAM, CTNNB1, PRX, GJA3, LSAMP, and AQP5 all demonstrating a lower percentage of the protein being found in the WI fraction in old samples compared to young samples, with the OWI versus YWI comparison being significant (adjusted *P* < 0.01) for all of these (Table 1B).

**Table 1.** Differences in the Top 15 Most Abundant More Soluble and More Insoluble Proteins

Protein	ID	YWS	YWI	OWS	OWI	Young %Insoluble	Old %Insoluble
A. Top 15 relatively more soluble proteins
Crystallin beta B1	CRYBB1	1023	203	1348	302	16.6%	18.3%
Crystallin beta B2	CRYBB2	826	276	859	269	25.0%	23.8%
Crystallin beta A1	CRYBA1	673	157	808	237	18.9%	22.7%
Gamma-crystallin C	CRYGC	814	141	819	147	14.8%	15.2%
Crystallin gamma S	CRYGS	550	110	1008	179	16.7%	15.1%
Betaine–homocysteine S-methyltransferase 2/Crystallin psi	BHMT2	435	142	494	206	24.6%	29.4%
Crystallin beta A4	CRYBA4	396	96	493	145	19.5%	22.7%
Crystallin gamma D	CRYGD	375	73	524	118	16.3%	18.4%
Crystallin beta B3	CRYBB3	302	117	327	106	27.9%	24.5%
Serpin family B member 6	SERPINB6	216	45	333	78	17.2%	19.0%
Crystallin gamma B	CRYGB	240	45	295	65	15.8%	18.1%
Aldehyde dehydrogenase 1 family member A1	ALDH1A1	102	40	143	82	28.2%	36.4%
Beta/gamma crystallin “Greek key” domain-containing protein (crystallin gamma A)	LOC711450 (CRYGA)	131	22	110	24	14.4%	17.9%
Peroxiredoxin-6	PRDX6	79	34	77	41	30.1%	34.7%
Protein deglycase	PARK7	36	6.3	68	11	14.3%	13.9%
Fatty acid binding protein 5	FABP5	57	12	33	12	17.4%	26.7%
B. Top 15 relatively more insoluble proteins							
Beaded filament structural protein 2	BFSP2	37	612	43	420	94.3%	90.7%
Filensin	BFSP1	48	595	53	391	92.5%	88.1%
Plectin	PLEC	14	83	20	57	85.7%	73.8%
Gap junction protein	GJA8	1.5	83	1.0	33	98.2%	97.1%
Solute carrier family 2, facilitated glucose transporter member 1	SLC2A1	0.3	25	0.3	17	98.8%	98.2%
Neuronal cell adhesion molecule	NRCAM	0.3	26	0.3	8.0	98.9%	96.4%
Catenin beta-1	CTNNB1	2.8	17	2.3	9.6	86.3%	80.7%
Periaxin	PRX	0.6	20	0.3	7.6	97.1%	96.2%
Gap junction protein	GJA3	0.3	17	0.3	7.8	98.3%	96.3%
Limbic system associated membrane protein	LSAMP	0.3	18	0.3	6.6	98.3%	95.7%
Erythrocyte membrane protein band 4.1 like 2	EPB41L2	2.2	13	1.0	8.2	85.1%	89.1%
Glucose-6-phosphate 1-dehydrogenase	G6PD	3.1	4.3	3.9	12	58.1%	75.0%
Cofilin-2	CFL2	1.6	9.9	1.0	8.1	86.1%	89.0%
Aquaporin-5	AQP5	0.3	9.3	0.3	8.4	96.9%	96.6%
Lactase like	LCTL	0.6	10	0.3	6.7	94.4%	95.7%

TMM-normalized average protein SpC values are shown for each group. Proteins are abundance ranked based on mean SpC of all samples. The percentage of the protein found in the water-insoluble fraction by age was calculated based upon the mean SpC of each group. Adjusted *P* values were less than 0.01 for all proteins except G6PD, which was not statistically significant when comparing OWI to OWS.

Proteins that lose their normal solubility with advancing age are of the greatest interest, as these differences are likely to play a role in the development of age-related nuclear cataract. To assess this subset of lens proteins, all proteins with a significant difference in the OWI versus YWI or OWS versus YWS comparison were selected and the percentage of the protein found in the insoluble fraction was calculated from the mean spectral count of each group. This set of proteins was then ordered by the differences between the percentage insoluble in old lens and the percentage insoluble in young lens. The top 30 of these proteins are listed in Table 2. This list includes many proteins involved in glycolysis, including G6PD, GAPDH, AKR1B10, ALDOC, PKM, PGK1, ALDOA, and PFKL. Other genes involved in linking other energy sources to glycolysis, including GALM and SORD, were also listed. Enzymes related to the essential antioxidant glutathione (GSH), ADHX, and GSS, also showed an age-related difference in solubility. As expected, several beta and gamma crystallins demonstrated an age-related difference in solubility.

**Table 2.** Top 30 Proteins with Age-Related Differences in Solubility

Protein	ID	YWS	YWI	OWS	OWI	Young %Insoluble	Old %Insoluble
F-box only protein 17	FBXO17	1.9	0.2	2.4	1.8	8.9%	43.2%
Rab GDP dissociation inhibitor	GDI1	7.4	4.2	4.9	9.3	35.9%	65.4%
Malic enzyme	ME1	6.3	0.9	8.0	5.2	12.3%	39.4%
Rab GDP dissociation inhibitor	GDI2	14	7.9	13	21	36.4%	61.3%
Carbonyl reductase (NADPH)	CBR1	5.8	2.7	6.4	7.2	32.0%	52.8%
S-(hydroxymethyl)glutathione dehydrogenase	ADH5	5.9	2.5	6.4	6.3	29.6%	49.8%
Aldo-keto reductase family 1 member B	AKR1B1	6.3	0.5	9.0	3.2	8.1%	26.1%
WD repeat domain 1	WDR1	3.4	3.8	3.1	7.2	52.5%	69.8%
Glucose-6-phosphate 1-dehydrogenase	G6PD	3.1	4.3	3.9	12	58.6%	75.0%
Glyceraldehyde-3-phosphate dehydrogenase	GAPDH	114	51	76	65	31.1%	45.9%
Aldo-keto reductase family 1 member B10	AKR1B10	1.5	0.5	5.6	3.9	26.4%	40.8%
Fructose-bisphosphate aldolase	ALDOC	23	11	30	25	30.9%	45.1%
Nucleoside diphosphate kinase	NME2	11	0.4	19	3.6	3.1%	16.0%
Tubulin alpha chain	TUBA1C	30	21	34	41	42.0%	54.8%
Coactosin like F-actin binding protein 1	COTL1	9.0	6.9	12	15	43.4%	56.0%
Aldose 1-epimerase	GALM	0.7	0.3	2.7	2.4	34.7%	46.6%
Glutathione synthetase	GSS	30	6.7	35	15	18.3%	29.9%
Pyruvate kinase	PKM	17	18	16	26	50.4%	61.5%
Crystallin beta A2	CRYBA2	74	44	30	27	37.2%	47.9%
Fatty acid binding protein 5	FABP5	57	12	33	12	17.6%	26.8%
Aldehyde dehydrogenase 1 family member A1	ALDH1A1	103	40	143	82	28.3%	36.5%
Phosphoglycerate kinase	PGK1	29	6	34	12	17.8%	25.5%
Crystallin gamma A	CRYGA	39	27	20	18	40.5%	47.2%
Fructose-bisphosphate aldolase	ALDOA	12	6.9	15	12	37.3%	42.9%
Transketolase	TKT	25	4.5	44	11	15.2%	20.7%
Sorbitol dehydrogenase	SORD	19	14	28	26	43.5%	47.7%
Crystallin beta A1	CRYBA1	673	157	808	237	18.9%	22.7%
ATP-dependent 6-phosphofructokinase	PFKL	0.6	1.4	2.3	6.3	70.2%	73.5%
Crystallin beta A4	CRYBA4	396	97	493	145	19.6%	22.7%
Crystallin gamma D	CRYGD	375	73	524	119	16.3%	18.4%

Mean SpC are given and used for calculation of percentages. All proteins had significantly different (adjusted *P* < 0.01) abundance in the OWI versus YWI or OWS versus YWS comparison, or both. Proteins were ranked by the difference in percentage of insolubility between old and young.

### Comparison of Rhesus Macaque Lens Proteome to Human Lens Proteome

Features of the WS fraction of the 4-year old (young) rhesus macaque lens nucleus proteome, such as the portion of the proteome consisting of crystallins and the relative amount of each family of crystallins, were compared to that of the WS fraction of a 3-day-old human lens using data from a prior study^28^ in order to determine similarity between the proteomes (Table 3). This study was chosen for comparison because it is the closest available dataset to the present study in terms of lens processing, LC separation, mass spectrometer used, data acquisition method, and data analysis pipeline. Additionally, data from 5-day old, 23-day old, and 18-month old human lenses from a study on the absolute abundance of human lens crystallins was included but data on the beaded filament and other protein fractions of these lenses were not available.^29^ Although the rhesus macaque data are from lens nucleus only and the human data are from whole lenses, these are largely equivalent cell populations, as fiber cells that make up the infant lens will become the lens nucleus in adolescence and adulthood.

**Table 3.** Comparison of Crystallin and Filament Proteins of the Water-Soluble Fractions of 4-Year-Old Rhesus Macaque Lenses and Infant Human Lenses

Feature	Rhesus Macaque 4-Y WS Mean	Human 3-D WS^28^	Human 5-D WS Mean^29^	Human 23-D WS Mean^29^	Human 18-Mo WS Mean^29^
Crystallin fraction of proteome	80.2%	84.2%	–	–	–
Alpha fraction of crystallins	27.0%	27.3%	33.2%	31.5%	32.3%
Alpha A to alpha B ratio	1.73	2.10	4.54	4.89	2.84
Beta fraction of crystallins	42.5%	54.0%	41.6%	40.6%	43.2%
Acidic betas fraction	14.2%	19.3%	14.0%	13.7%	12.7%
Basic betas fraction	28.3%	34.7%	27.5%	27.0%	30.5%
Basic to acidic ratio	1.99	1.88	1.96	1.98	2.40
Gamma fraction of crystallins	25.1%	18.6%	25.5%	27.8%	24.5%
Gamma S fraction	6.8%	8.8%	7.0%	6.7%	8.0%
Other gammas fraction	18.3%	9.8%	18.5%	21.1%	16.4%
Gammas to gamma S ratio	2.68	1.11	2.63	3.14	2.04
Beta + gamma fraction of crystallins	67.5%	72.7%	67.1%	68.5%	67.7%
Taxon-specific fraction	5.4%	0.0%	0.0%	0.0%	0.0%
Beaded filament fraction of proteome	1.1%	1.2%	–	–	–

Mean SpC from Wilmarth et al.^28^ and absolute abundance measurements from Halverson-Kolkind et al.^29^ were used for the calculation of the percentages and ratios.

The crystallin and beaded filament fractions were very similar between species, respectively making up 84.2% and 1.2% in 3-day old human lens and 80.2% and 1.1% in 4-year-old rhesus macaque lens nuclei. The relative proportions of crystallin families and subgroups were also highly similar between species, with beta crystallins making up 42.5% of the crystallin fraction in young rhesus macaque lens nuclei and 40.6% to 54.0% in young human lenses. The ratio of basic to acidic beta crystallins was very similar, being 1.99 in macaques and 1.88 to 2.40 in humans. Gamma crystallins made up 25.1% of the total in macaques and 18.6% to 27.8% in humans. The ratio of other gamma crystallins to gamma S crystallin in macaques, 2.68, also fell within the human range of 1.11 to 3.14. Considered as a superfamily, the total portion of the crystallin fraction made up by beta and gamma crystallins together was quite similar at 67.5% in macaques and ranging from 67.1% to 72.7% in humans. Outside of the presence of the taxon-specific psi crystallin in the macaque lens, which made up 5.4% of the crystallin fraction while being completely absent from the human lens, the only area of divergence between species lies in the alpha crystallins. The fraction of total crystallins made up by the alpha family was slightly lower in macaques at 27.0%, compared to the range of 27.3 % to 33.2% in humans. However, if psi crystallin is not considered in the calculation, then the proportion of all crystallin families in macaques falls within the same range as humans, so the unique presence of this taxon-specific crystallin somewhat confounds the comparison. Regardless, the ratio of 1.73 for alpha A crystallin to alpha B crystallin in rhesus macaque lens nuclei was outside of the human lens range of 2.10 to 4.89.

### Global Analysis of Post-Translational Modifications

**Figure 2.**
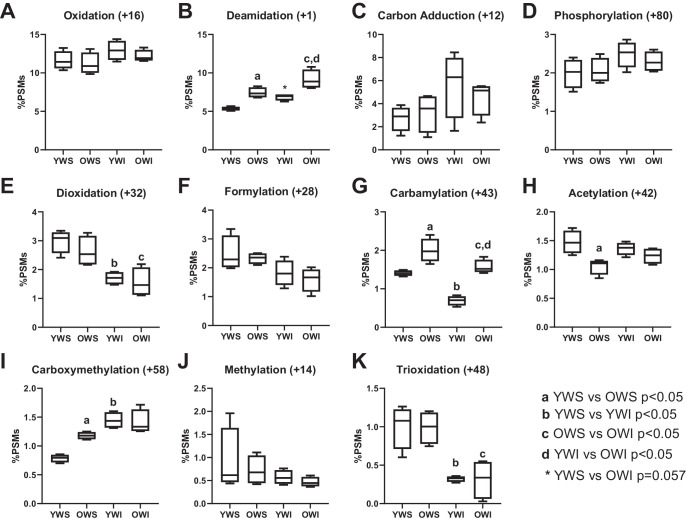
**Comparison of relative abundance of PTMs.** Box and whisker plots indicate the four quartiles and the median of each group. Titles of graphs indicate the common name of the PTM and its associated mass shift (to the nearest integer). All groups contained four each.

PTM-Shepherd was used to generate summary data from an open modification search of the proteomic dataset. The full set of summarized PTM data can be found in Supplementary Appendix S2. Figure 2 presents the comparison of percentages of detected PSMs bearing detected biologically relevant modifications found on at least 1% of PSMs in at least one sample group. Oxidation was the most abundant PTM and appeared to have a slight trend of increased frequency in the WI fraction, but this was not statistically significant (see Fig. 2A). Deamidation was the next most abundant PTM and showed differences based on both age and solubility, with YWS versus OWS, OWS versus OWI, and YWI versus OWI comparisons all being significant (*P* < 0.05) and the YWS versus YWI comparison being borderline significant (*P* = 0.0571; see Fig. 2B). The overall trend was higher %PSMs at the later age and higher %PSMs in the WI fraction compared to the WS fraction.

Carbon adduction was a notably abundant PTM that seemed to be slightly more prevalent in the WI fractions but was highly variable and comparisons did not reach the threshold of statistical significance (see Fig. 2C). Phosphorylation was found on approximately 2% of PSMs, with no significant differences found across sample groups (see Fig. 2D). Dioxidation appeared to be associated with solubility but not age, making up 3.0% of PSMs in YWS samples but only 1.7% in YWI samples (*P* < 0.01; see Fig. 2E). Similarly, this PTM was found on 2.6% of PSMs from OWS samples but only 1.6% of PSMs from OWI samples (*P* < 0.05). Formylation also appeared to show a trend of lower abundance in the WI fractions of both age groups, but this did not reach statistical significance (see Fig. 2F).

Carbamylation showed very clear differences based on both age and solubility (see Fig. 2G). The old lenses showed significantly higher levels of carbamylation in both fractions (*P* < 0.005). Within each age group, carbamylation was significantly more abundant in the WS fraction compared to the WI fraction (*P* < 0.05).

Acetylation was found at a slightly lower percentage in OWS compared to YWS (*P* < 0.01), with no significant differences in the WI fraction (see Fig. 2H). Carboxymethylation was significantly higher in OWS samples compared with YWS samples (*P* < 0.01) and significantly higher in YWI samples compared with YWS samples (*P* < 0.0001; see Fig. 2I). Methylation was a minorly abundant PTM found on less than 1% of PSMs on average with no significant differences between sample groups (see Fig. 2J). Trioxidation followed the same trend as dioxidation with decreased levels in the WI fractions compared with the WS fractions at both ages (*P* < 0.005; see Fig. 2K).

## Discussion

### Rhesus Macaque Lenses Display Age-Related Solubility Differences in Crystallins and Essential Enzymes

Because the lens nucleus lacks the synthesis machinery necessary for gene expression to occur,^5^ protein abundance differences between young and old lens nuclei are assumed to primarily be the result of either loss of solubility or degradation of the protein with age. A general trend of highly abundant cytosolic lens proteins (mostly crystallins) having reduced water-solubility with advanced age was noted in this study (see Table 1A). The opposite trend was also found in many of the most highly abundant naturally WI proteins (i.e. membrane-bound and cytoskeletal proteins), with a higher percentage of the protein found in the WS fraction at the more advanced age (see Table 1B). It is exceedingly unlikely that such proteins are shifting from the WI to the WS fraction with age, as such an occurrence has never been reported in the literature and has no clear mechanism. Rather, this may be a consequence of the median SpC data normalization method used in the preparation of these results. Because crystallins are so much more abundant than other proteins in the lens, their shift from WS to WI with age may decrease the median level of proteins normally in the WI fraction. Another potential explanation for this is that truncation of these proteins, a well-established PTM that occurs to lens proteins with age but not measured in this study,^2^ results in some soluble protein fragments that have been cleaved from these insoluble proteins entering the WS fraction whereas the rest of the protein remains in the WI fraction, therefore slightly altering the SpCs.

The set of proteins showing the greatest increase in percent found in the WI fraction in old compared with young samples consisted primarily of enzymes directly involved in or linked to glycolysis, as well as lens crystallins (see Table 2). The functional consequences of this are obvious, as mature lens fiber cells are reliant on anaerobic glycolysis to meet their energy needs and restriction of these pathways has been found to be causative for cataract formation in other models,^30^^,^^31^ whereas loss of solubility in lens crystallins is considered to be the major driver of age-related nuclear cataract.^3^^,^^4^ Notably, two enzymes related to the essential antioxidant GSH also showed significant age-related differences in solubility: glutathione synthetase (GSS) and S-(hydroxymethyl)glutathione dehydrogenase or alcohol dehydrogenase class III (ADHX). The former enzyme catalyzes the first step of GSH biosynthesis, whereas the latter is involved in the GSH-dependent detoxification of cellular formaldehydes. Deficiency in lens GSH has been demonstrated to contribute to cataract formation.^32^^,^^33^ Thus, most proteins associated with the age-related solubility differences detected in this study can be linked to disruption of lens homeostasis and cataractogenesis.

### The Rhesus Macaque Lens Proteome Recapitulates the Human Lens Proteome, With a Few Notable Distinctions

The overall composition of major lens proteins in the 4-year-old rhesus macaque lens nucleus closely matched that of a 3-day-old human lens (see Table 3). Additionally, when analyzing crystallins specifically, there was close similarity between the proportions of the different families and subgroupings found in the 4-year-old rhesus macaque lens nucleus and the 3-day-old, 5-day-old, 23-day-old, and 18-month-old human lenses. One major distinction is the previously noted presence of BHMT2 in rhesus macaque lenses, which has also been termed psi crystallin^15^ and makes up 5.4% of the total crystallin fraction of these lens nuclei. This enzyme was the sixth most abundant protein in the rhesus macaque lens nucleus but is completely absent from the human lens. Psi crystallin could potentially alter cytosol dynamics but, unfortunately, the role of this enzyme in the rhesus macaque lens has not been widely studied. The ratio of alpha A to alpha B crystallin was also somewhat lower in rhesus macaques compared with the human samples. It should be noted that this comparison is not perfect given that a 4-year-old macaque is approximately equivalent to a 16-year-old human in terms of lifespan progression. Therefore, data from a teenage human lens nucleus would be a more ideal comparison for this study, but such a dataset was not available. Therefore, whole lenses from human infants were the closest available comparison.

### Rhesus Macaque Lens Nuclei Demonstrate Age-Related Differences in Deamidation, Carbamylation, and Carboxymethylation, With Limited Differences in Other PTMs

Single oxidation was the most abundant PTM in the rhesus macaque lens (see Fig. 2A). Oxidation of lens proteins has long been associated with age-related cataract and is thought to be produced primarily from UV light exposure.^34^^,^^35^ However, it can be difficult to determine the biological relevance of this modification given that methionine oxidation can also be induced by sample processing and ionization steps.^36^ This may result in masking true differences between groups and prevents any definitive conclusions regarding this PTM. Dioxidation and trioxidation are less likely to be artifactual in nature and were found at significantly higher levels in the WS fraction compared to the WI fraction for both young and old lenses (see Fig. 2E, 2K). The reason for this remains unclear given that it is very unlikely that dioxidation or trioxidation enhances protein solubility. It is possible that residues on proteins found in the WI fraction are inherently less available to be oxidized as they may be embedded within membranes or insoluble aggregates where oxidizing agents may have a harder time reaching them. Therefore, they are less likely to reach a di- or tri-oxidized status.

Deamidation of asparagine to aspartic acid and glutamine to glutamic acid has been long established as a major PTM of the aging lens in humans and other model organisms^2^^,^^6^ and was also an abundant PTM in the rhesus macaque lenses (see Fig. 2B). Deamidation was found preferentially in the WI fraction of both young and old lenses, indicating the expected loss of solubility associated with this modification. Additionally, deamidation was significantly age-associated, as has been reported in other studies.^19^ This provides further evidence of the rhesus macaque lens closely mimicking the biology of the human lens. However, it should be noted that correctly distinguishing between deamidation and precursor isotopic peaks in bottom-up proteomics continues to be challenging even with high resolution, high mass accuracy instrumentation.

Carbon adduction results from the addition of a single carbon to cysteine, lysine, or tryptophan that can form from electrophilic attack of formaldehyde.^37^ This PTM was found at a high abundance in the macaque lens, although with a high level of variance and no statistically significant differences between groups (Fig. 2C). These results are somewhat surprising given the lack of reported characterization of this PTM in lens proteins. Formaldehyde can exist as an environmental hazard but is also a ubiquitous endogenous metabolite formed as a by-product of enzymatic oxidative demethylation reactions.^38^ Endogenous formaldehyde reacts with GSH, an antioxidant peptide that is highly abundant in the lens, to form S-hydroxymethyl-GSH, which is then broken down to formate by the enzyme S-(hydroxymethyl)glutathione dehydrogenase, also known as ADHX.^38^ The concentration of GSH in the lens nucleus has long been known to diminish with age due to the formation of a barrier to its diffusion to the center of the lens.^33^^,^^39^ Given GSH's known role in protecting against cataract formation, this gives carbon adduction of lens proteins via formaldehyde a clear potential role in cataractogenesis as the levels of this damaging modification would be expected to rise as GSH levels decrease, perhaps further exacerbated by the decrease in GSS solubility at older age noted in this study. Furthermore, ADHX was detected in our proteomic analysis of these lenses and had one of the largest age-related differences in solubility with 29.6% of the protein being WI in young lenses but 49.8% WI in old lenses. Further research is required to follow up on these intriguing results and elucidate the role of carbon adduction, ADHX, and their interplay with GSH dynamics in lens aging and cataract formation.

Phosphorylation is another well-characterized PTM of the lens. Phosphorylation on alpha crystallins has been reported to have both positive and negative effects on solubility depending on the specific residue and the degree of phosphorylation across the protein,^40^^,^^41^ whereas little has been reported on the effects of phosphorylation on beta/gamma crystallins. Given the enzymatic and site-specific nature of phosphorylation, it is unsurprising that no significant differences were noted at the global level in this analysis.

Formylation is a PTM primarily associated with histone proteins, where it arises as a secondary result of oxidative DNA damage.^42^ To date, no other studies appear to have characterized formylation as a PTM of the lens and the biological mechanism by which it would occur is unclear given that mature lens fiber cells lack DNA. Formic acid used in liquid chromatography buffers, including this study, is a potential artifactual source of formylation.

Carbamylation is a damaging nonenzymatic modification that can occur in the presence of high levels of urea which dissociates to isocyanate that binds lysine residues. Carbamylation has been found to be highly associated with aging^43^ and has been detected on lens crystallins previously, where it causes alterations to protein structure.^44^^,^^45^ The source of lens carbamylation is presumably the endogenous production of urea from amino acid metabolism in the lens and surrounding tissues as no urea was used in sample preparation. In this study, carbamylation was found to be at significantly higher levels in old lenses compared with young lenses for both fractions (see Fig. 2G). Carbamylation was also significantly more abundant in the WS fraction compared to the WI fraction at both ages, similar to dioxidation.

Acetylation from lysine acetyltransferases and N-terminal protein processing is another well-established PTM of the human lens.^6^ Studies have indicated that this PTM may alter crystallin structure, solubility, and chaperone function,^46^ but it appears that these changes may actually enhance the water solubility of crystallins and reduce lens stiffness.^47^ Acetylation was found to be at a significantly lower level in OWS samples compared with YWS samples in rhesus macaque lenses (see Fig. 2H). This loss of acetylation status could contribute to the loss of lens protein solubility with age.

Carboxymethylation is an advanced glycation end-product (AGE) and a well-characterized PTM of the lens that forms on lysine residues with age via the Maillard reaction.^48^^,^^49^ This carboxymethylation is believed to impact protein structure, function, and solubility, contributing to cataract formation.^19^ In this study, carboxymethylation was found to be significantly higher in OWS versus YWS and significantly higher in YWI versus YWS (see Fig. 2I). This follows the expected result that carboxymethylation increases with age and is associated with loss of solubility.

Methylation is another common PTM found in lenses, believed to be caused by nonenzymatic attack from S-adenosylmethionine (SAM) on lysine residues.^50^ However, the actual impact of methylation on lens proteins remains relatively uncharacterized. In this study, there appeared to be trends of lower methylation levels at older age and in the WI fraction compared to the WS fraction, but these did not reach the threshold of significance (see Fig. 2J).

Overall, these PTM results provide further basis for the rhesus macaque lens acting as an effective model for the study of human cataractogenesis, given the presence of many of the same PTMs characterized in the human lens which appear to follow similar solubility and aging trends. At the same time, these data provide intriguing new avenues for research with the detection of high levels of carbon adduction and formylation in these lenses not previously characterized in other studies.

### Limitations

There are important limitations to this study that are worth considering. The lens cortex tissue was not included in this lens nucleus study, so age-related regional differences in the lens were not assessed. Whereas we believe that that the four-fold age difference between the young and old animals used in this study is adequate to assess the age-related differences in the lens proteome, it is true that comparing even younger tissue to even older tissue would very likely draw out more distinct proteomic differences. There is also the possibility of different trends in lenses from male animals as only tissue from female animals was used in this study. Only the 201 most abundant proteins were quantified in our analysis. Many other proteins are known to be present in the lens but were outside of the dynamic range of quantitation and therefore not presented to avoid any false conclusions or misinterpretation.

Additionally, although we have assessed a variety of PTMs, there are many more PTMs that our methodology did not allow us to probe. For example, truncation, racemization, glutathionylation, and crosslinking of proteins are known to be common effects of aging on the lens^2^ and were not analyzed in this study due to the need for specialized methodologies to measure these modifications rather than the generalized approach used here. The list of PTMs presented here should not be taken to be complete or comprehensive. We have also only assessed PTMs at a global level rather than at the level of individual peptides. There were not enough individual modified peptides that were detected across a majority of the samples to provide the resolution necessary to confidently report peptide-level results. Whereas PTMs such as methylation and formylation did not show significant differences when analyzed in bulk, there could be important site-specific differences at the peptides level which may significantly impact protein solubility, function, and play major roles in age-related lens pathology. Methodology tailored to analyzing specific PTMs, rather than the more general approach we have taken here, is necessary to produce high confidence data on site-specific PTM differences.

## Conclusions

The rhesus macaque lens proteome shows strong similarity to that of the human lens, with a similar proportion of protein types and crystallin groups, the same trends in protein solubility differences at older ages, and many of the same PTMs present. Thus, the rhesus macaque could be a useful animal model for studying the effects of age on lens proteins. However, some of the unique features of the rhesus macaque lens proteome, which include the presence of high levels of betaine-homocysteine S-methyltransferase 2/psi crystallin and relatively high levels of carbon adduction and formylation, should also be considered.

## Supplementary Material

Supplement 1

Supplement 2

Supplement 3
