# Supplementary material for: Proteomic Characterization of the Rhesus Macaque Lens Nucleus: Similarity to Human Lens, Age Effects on Protein Solubility, and Trends in Post-Translational Modifications
Source: Invest Ophthalmol Vis Sci. 2025 Sep 12;66(12):28. doi: 10.1167/iovs.66.12.28 (PMC12439503; doi:10.1167/iovs.66.12.28)

**Supplemental Table 1. HP-LC Settings.**

Dionex NCS-3500RS UltiMate Settings: Software Version: Thermo SII 1.5.0.10747

|  |  |  |
| --- | --- | --- |
| Buffer Info |  |  |
|  | Mobile Phase A | 0.1% Formic Acid in Water |
|  | Mobile Phase B | 0.1% Formic Acid in Acetonitrile |
|  | Loading Time | 5 minutes |
| HPLC |  |  |
|  | Trap Column | Thermo Acclaim PepMap C18 100 µm x 2 cm NanoViper, 5 µm |
|  | Analytical Column | Thermo PepMap RSLC C18, 75 µm x 25 cm EasySpray, 2 µm |
|  | Flow Rate | 600 nL/min |
|  | Run Time | 90 min |
|  | Loading Mobile Phase | 100% Mobile Phase A |
| Gradient Profile |  |  |
|  | Time | Mobile Phase B Composition |
|  | 0.0-5.0 min | 2% |
|  | 5.0-5.1 min | 7.5% |
|  | 5.1-65.0 min | 30% |
|  | 65.0-66.0 min | 98% |
|  | 66.0-71.0 min | 98% |
|  | 71.0-72.0 min | 2% |
|  | 72.0-90.0 min | 2% |

**Supplemental Table 1. Mass Spectrometry Settings.**

Orbitrap Q-Exactive Settings: Instrument control software version 3.4.3072.18

|  |  |  |
| --- | --- | --- |
| General Settings |  |  |
|  | Method duration | 90 min |
|  | Ion source type | NSI |
|  | Spray voltage positive ion | 2,400 V |
|  | Ion transfer tube temperature | 300 C |
|  | Use Lock Masses | Best |
| MS1 Scans |  |  |
|  | Detector | Orbitrap |
|  | MS1 resolution | 120,000 |
|  | Scan range | 375 to 1,400 m/z |
|  | Spectrum Data | profile |
|  | Maximum inject time | 100 ms |
|  | AGC target | 3e6 |
| Dynamic exclusion settings |  |  |
|  | Duration Exclusion | Auto |
|  | Charge Exclusion | Unassigned, 1, 8, >8 |
|  | Minimum AGC Target | 5.0e3 |
|  | Exclude isotopes | on |
|  | Peptide Match | preferred |
| MS2 Scans |  |  |
|  | Detector | Orbitrap |
|  | Isolation Window | 1.2 m/z, no offset |
|  | Fragmentation | HCD |
|  | Normalized collision energy | 30 % |
|  | Maximum IT | 100 ms |
|  | Resolution | 30,000 |
|  | Scan range | 200 to 2,000 m/z |
|  | Fixed First Mass | 100 m/z |
|  | AGC target | 1e5 |
|  | Spectrum Data | centroid |
|  | Loop Count | 10 |


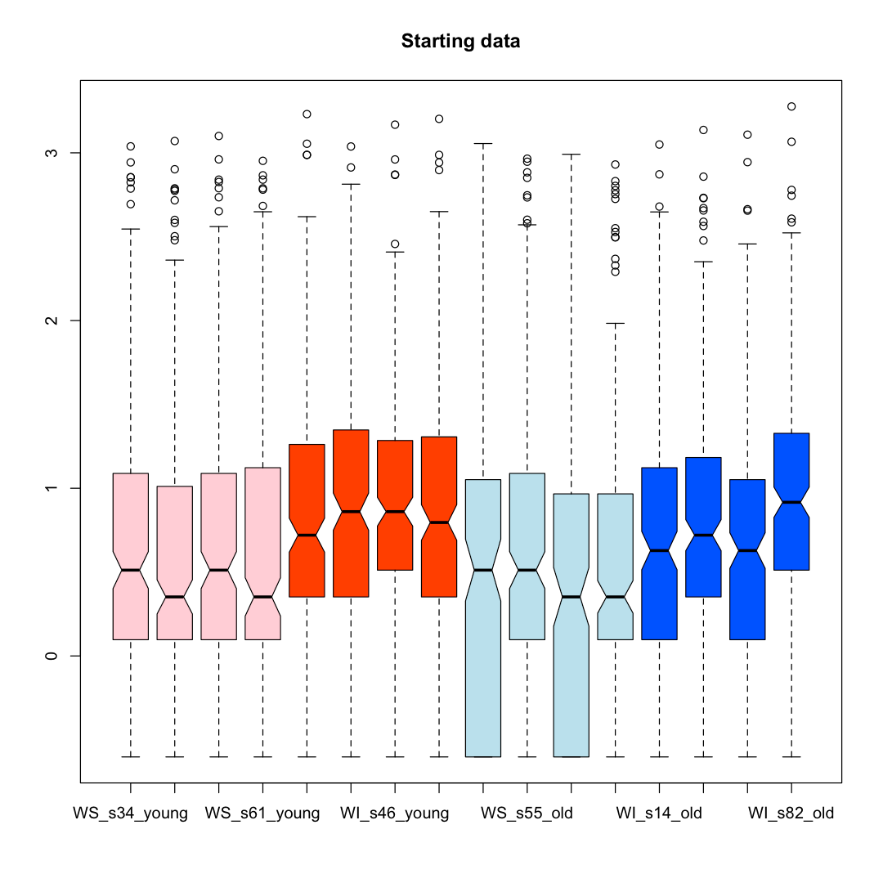


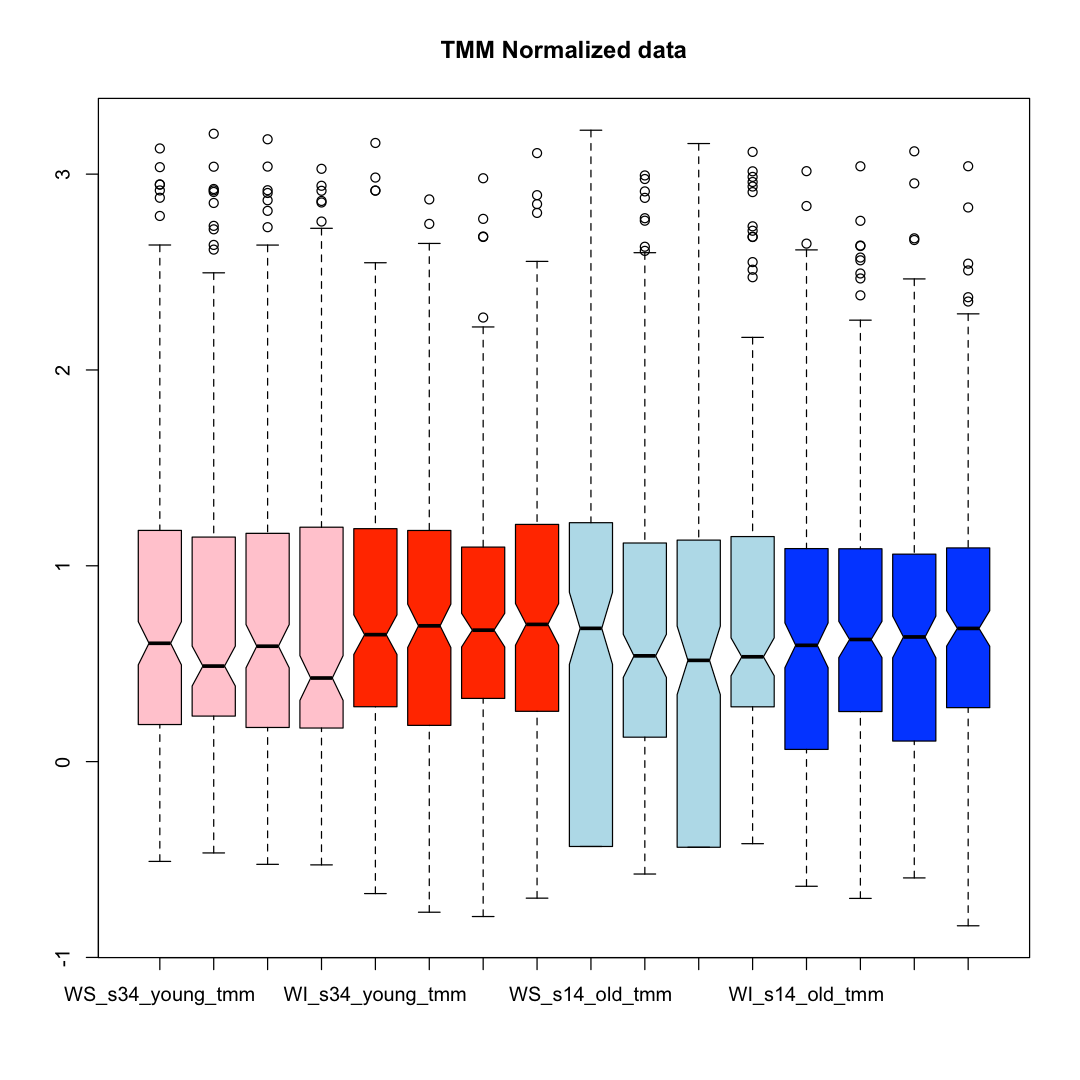


**Supplemental Figure 1. Boxplot Visualization of Protein Abundance Data Before (Top) and After (Bottom) TMM Normalization.**
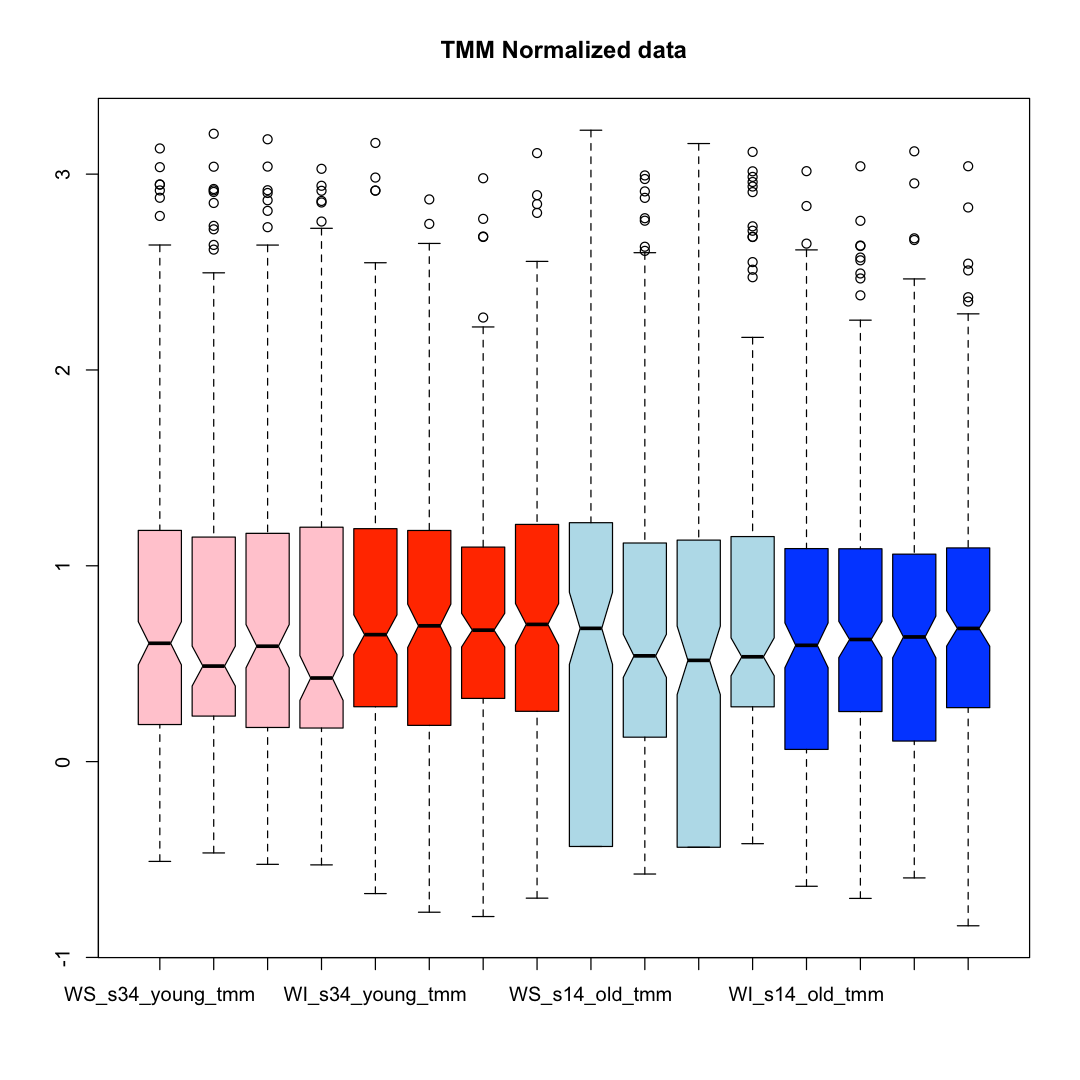

Supplement: Supplement 1 [file iovs-66-12-28_s001.docx]
